# Combined thermal and mild electrical stimulations modulate heat shock protein and VEGF in retinal pigment epithelial cells under high glucose

**DOI:** 10.1186/s13104-025-07539-y

**Published:** 2025-10-29

**Authors:** Ryosuke Fujino, Kiyoto Totsuka, Tomoyasu Shiraya, Takashi Ueta, Fumiyuki Araki, Ryo Terao

**Affiliations:** 1 Department of Ophthalmology, Graduate School of Medicine, the University of Tokyo, Tokyo, Japan; 2 Department of Ophthalmology, Showa General Hospital, Tokyo, Japan

**Keywords:** Diabetic macular edema, Heat shock protein, Thermal stimulation, Mild electrical stimulation

## Abstract

**Objective:**

Macular laser for diabetic macular edema (DME) is known to increase heat shock protein (HSP) expression in retinal pigment epithelial (RPE) cells, and increased HSP expression may be a mechanism for improving macular edema. Furthermore, the effectiveness of mild electrical stimulation (MES) as a cofactor in further enhancing HSP expression by thermal stimulation has been reported. In the current study, the effect of this combination treatment was examined in an in vitro HG (high glucose) + DFX (deferoxamine mesylate salt) model that simulates DME using a human RPE cell line (ARPE-19).

**Results:**

Combined thermal stimulation and MES significantly increased HSP70 expression in both the control and HG + DFX model groups, while suppressing VEGF and inflammatory cytokine levels in HG + DFX model. The combined treatment also showed a protective effect on the blood-retinal barrier integrity, although transient, as measured by TEER. These findings suggest that combined thermal stimulation and MES represent a promising therapeutic approach for managing DME, by modulating HSP and VEGF expression and potentially reducing inflammation and promoting protective mechanisms in the retina.

**Supplementary Information:**

The online version contains supplementary material available at 10.1186/s13104-025-07539-y.

## Introduction

Diabetic retinopathy (DR) and diabetic macular edema (DME) are serious complications of diabetes that can lead to significant vision loss [1]. The pathogenesis of these conditions involves a complex interplay of factors including hyperglycemia, hyperlipidemia, hypoxia, and reactive oxygen species [2]. Current treatment for DR and DME primarily focuses on anti-vascular endothelial growth factor (VEGF) drugs, laser therapy, and surgery [3]. Intravitreal anti-VEGF therapy has emerged as a promising treatment for DR and DME [4]. The treatment efficacy of anti-VEGF agents has been shown in clinical trials [5]. While effective, this invasive procedure carries potential risks, including endophthalmitis and retinal detachment. Moreover, anti-VEGF therapy requires repeated and frequent injections and incurs a significant financial burden. In fact, up to 50% of eyes treated with monthly anti-VEGF therapy have persistent DME and require alternative treatment [6].

Micropulse lasers have been developed to overcome the disadvantages of conventional macular grid lasers, such as atrophic creep, emergence of scotoma due to heat-induced destruction of the retina, and micropulse laser therapy alone or in combination with anti-VEGF therapy was considered a treatment for DME [7]. However, even micropulse laser therapy have limited therapeutic efficacy, and there are concerns about the mechanical or thermal damage in retina by the increased laser power.

While the mechanism of response to micropulse laser therapy remains unclear, the key trigger was an increase in heat shock protein (HSP) expression due to the thermal effect [8, 9]. HSP family, which acts as chaperone proteins, assists in the refolding of denatured proteins and is protective against apoptosis and inflammation [10–14]. In particular, thermal effects of laser irradiation are thought to upregulate HSP70 expression from RPE cells [15, 16]. Other previous reports have reported the potential of HSPs as therapeutic agents for DR [17].

Electrophysical therapy, including electrical stimulation, has also shown promise in managing diabetic complications. Studies have found that electrical stimulation can be beneficial for diabetic foot ulcers and improve pain relief in patients with diabetic neuropathy [18, 19]. Furthermore, studies using rat skeletal muscle cells and the liver of diabetic mouse models have reported mild electrical stimulation (MES) as a cofactor that further enhances HSP expression [20–22].

Therefore, we hypothesized that combined thermal stimulation and MES therapy may be useful in the treatment of DR and DME. Given that the therapeutic effects of subthreshold micropulse laser arise from its thermal effect on RPE cells, without directly affecting other retinal cell types [8, 9], we investigated the effects of combined thermal stimulation and MES on HSP expression, VEGF, and inflammatory cytokine levels in HG (high glucose) + DFX (deferoxamine mesylate salt) models that simulate the pathology of DME induced by hyperglycemia and hypoxia to determine its potential as a new therapeutic approach for the treatment of DR and DME.

## Methods

### Cell culture and treatment

ARPE-19 cells, a human RPE cell line, were used (#CRL-2303; American Type Culture Collection, VA, US). Cells were cultured in Dulbecco’s modified Eagle’s medium (DMEM; Thermo Fisher Scientific, MA, US) containing 10% fetal bovine serum (FBS) and an antibiotic–antimycotic solution (Sigma-Aldrich, MO, US) in a humidified 5% CO2 atmosphere at 37℃. Cells were passaged every 3–4 days using a trypsin-EDTA solution.

Cells were cultured in high-glucose medium (4.5 g/L; D-MEM High Glucose, FUJIFILM Wako Pure Chemical Corporation., OS, JP) and DFX (100 µM; D9533; Sigma-Aldrich, MO, US) for 24 h as an in vitro model of DME based on previous a previous report [23]. For the control group, cells were cultured under normal glucose conditions (1 g/L; D-MEM Low Glucose, FUJIFILM Wako Pure Chemical Corporation., OS, JP).

### Heat shock therapy (HS) with mild electrical stimulation treatment (MES)

ARPE-19 cells were plated on 60 mm culture dishes, and at 80% confluency were subjected to a combined heat shock therapy (HS) and MES protocol as previously described [21, 24]. For MES, the rubber electrodes (KAITO DENSHI CO., LTD., ST, JP) were fitted at the walls of the culture plate and in contact with the culture media (Additional Figure S1). The electrodes were connected to a P4K36-1-LDe device (MATSUSADA precision inc., TY, JP). MES was applied using at a voltage of 5 V, with a pulse width of 1 s, followed by a 1-second interval, for a total of 300 pulses. The culture plate with the electrodes was carefully immersed in a water bath at a temperature of 42℃.

### Cytotoxicity assay

After cells were treated with HS + MES for 10 min, cell culture supernatants were collected 24 h after the treatment. Lactate dehydrogenase (LDH) activity in the cell culture supernatants was measured using the Cytotoxicity LDH Assay Kit-WST (DOJINDO LABORATORIES, KU, JP) following the manufacturer’s instructions. The percentage of cytotoxicity was calculated for each group using the following equation:

Cytotoxicity (%) = (Test Substance - Low Control) / (High Control - Low Control) x 100.

The cytotoxicity rates of each group were compared.

### Quantitative real-time polymerase chain reaction (qRT-PCR) assay

Total RNA was extracted from cells using TRI Reagent (Molecular Research Center, Inc., OH, US) 24 h and 48 h after the HS + MES treatment, and mRNA was isolated using chloroform and isopropyl alcohol. cDNA was synthesized from mRNA using a PrimeScript RT Reagent Kit (Takara Bio, SI, JP). qPCR was performed using the Thermal Cycler Dice Real Time System II (Takara Bio, SI, JP) with SYBR Premix Ex TaqII (Tli RNaseH Plus; Takara Bio, SI, JP). Primer sequences were obtained from previously published sequences and purchased from Hokkaido System Science (HK, JP). The primer sequences for the target genes and the housekeeping gene *β-actin* are shown in Additional Table S1. qPCR was performed using the ΔΔCt method, and target gene expression was normalized relative to that of *β-actin* mRNA. All tests were conducted in triplicate to ensure reproducibility, and the consistency of the experiment was confirmed using biological triplicates.

### Western blot assay

Cells were lysed in RIPA buffer (Thermo Fisher Scientific, MA, US) containing protease inhibitors (Roche Diagnostics, BS, CH) 6 h after HS + MES treatment. The lysate was centrifuged and the supernatant was collected as the protein extract. Protein concentration was determined using the BCA Protein Assay Kit (Thermo Fisher Scientific, MA, US) according to the manufacturer’s protocol using bovine serum albumin as a standard. Proteins were separated using 4 to 12% or 12% polyacrylamide gels and transferred to a polyvinylidene difluoride membrane (Bio-Rad Laboratories, CA, US). The membrane was blocked by Blocking One (Nacalai Tesque, Inc, KY, JP) for 10 min and then was incubated with primary antibody overnight at 4 °C. The images of the membrane were captured by an ImageQuant LAS 4000 mini-camera system (GE Healthcare, IL, US). The following primary antibodies were used: anti-HSP70 (1:100; Enzo Life Sciences, Farmingdale Railroad, NY, US) and anti-β-tubulin (1:10000; Wako Pure Chemical Industries, Ltd., OS, JP). For secondary antibodies, horseradish peroxidase-conjugated anti-mouse and anti-rabbit secondary antibody (1:2000 and 1:5000; Thermo Fisher Scientific, MA, US). The intensity of protein bands was quantified using ImageJ software (ver. 1.53, NIH, Bethesda, MD, US). Protein loading was normalized to β-tubulin expression.

### Enzyme-linked immunosorbent assay (ELISA)

VEGF levels in the cell culture supernatants were measured using the Human VEGF Quantikine ELISA Kit (R&D Systems, MN, US) according to the manufacturer’s instructions. Cell culture supernatants were collected at 24 h after HS + MES treatment. The concentration of VEGF in each sample was analyzed using a multimode microplate reader (2030 ARVO X3; Perkin Elmer, MA, US) at 450 nm with a wavelength correction of 570 nm. The results were calculated by constructing a standard curve.

### Measurement of transepithelial electrical resistance (TEER)

Epithelial barrier formation and polarization were determined by measurement of TEER. Cells were seeded in 12-well Transwell inserts at a density of 1 × 10^5^ cells/ml and cultured until confluent. TEER measurements were performed using a Millicell ERS-2 V-ohm meter (Merck Millipore, HE, DE) at 24 h and 48 h after HS + MES treatment. TEER values were calculated according to the previous study [25] and are given as Ω/cm2 and normalized to the mean. Each experiment was performed at least three times.

### Statistical analysis

Sample sizes were calculated using a priori power analysis (ClinCalc). Data points were excluded only when technical issues occurred, including contamination or assay failure. All experiments were performed in biological triplicates. Scatter plots are now shown to indicate individual data points and sample sizes for each group. All statistical analyses were performed using either the statistical programming language ‘R’ (version 4.0.3; R Foundation for Statistical Computing, WI, AT) or GraphPad Prism (version 10.6.0; GraphPad Software, CA, US). For comparisons involving two or more groups, one-way ANOVA was conducted, followed by an appropriate post-hoc test for multiple comparisons, such as the Tukey–Kramer test or the Bonferroni test. P-values less than 0.05 were considered statistically significant.

This manuscript was drafted and edited with the assistance of Google AI studio (Gemini 2.0 Flash Experimenta version; Google LLC, CA, US).

## Results

### Generation of HG + DFX model and cytotoxicity of HS + MES

In the retina, hyperglycemia impairs microvasculature, resulting in hypoxia and upregulation of VEGF, which contributes to the development of macular edema [23]. Deferoxamine (DFX) acts as a hypoxia mimetic by stabilizing hypoxia-inducible factor (HIF)-1α, thereby activating inflammatory and angiogenic pathways [26, 27]. Based on a previous report [23], we established an in DME model using HG and DFX (HG + DFX).

ARPE-19 cells were cultured in normal glucose (1 g/L) as controls or high glucose (4.5 g/L) + DFX (100 μm) as HG + DFX model that simulates DME for 24 h, followed by the treatment with HS + MES for 10 min. The mean (± standard deviation; SD) cytotoxicity of the control + HS + MES (*n* = 6) was − 9.9 ± 1.56%, HG + DFX model (*n* = 6) was − 18.0 ± 6.3%, and HG + DFX model + HS + MES (*n* = 6) was − 24.0 ± 1.1%. There was no significant difference in cytotoxicity across groups (*p* = 0.77, ANOVA), suggesting that HS + MES treatment and HG + DFX model did not have cytotoxic effects (Additional Figure S2).

### Effects of HS + MES on the expression of HSP70 in HG + DFX model

**Fig. 1:**
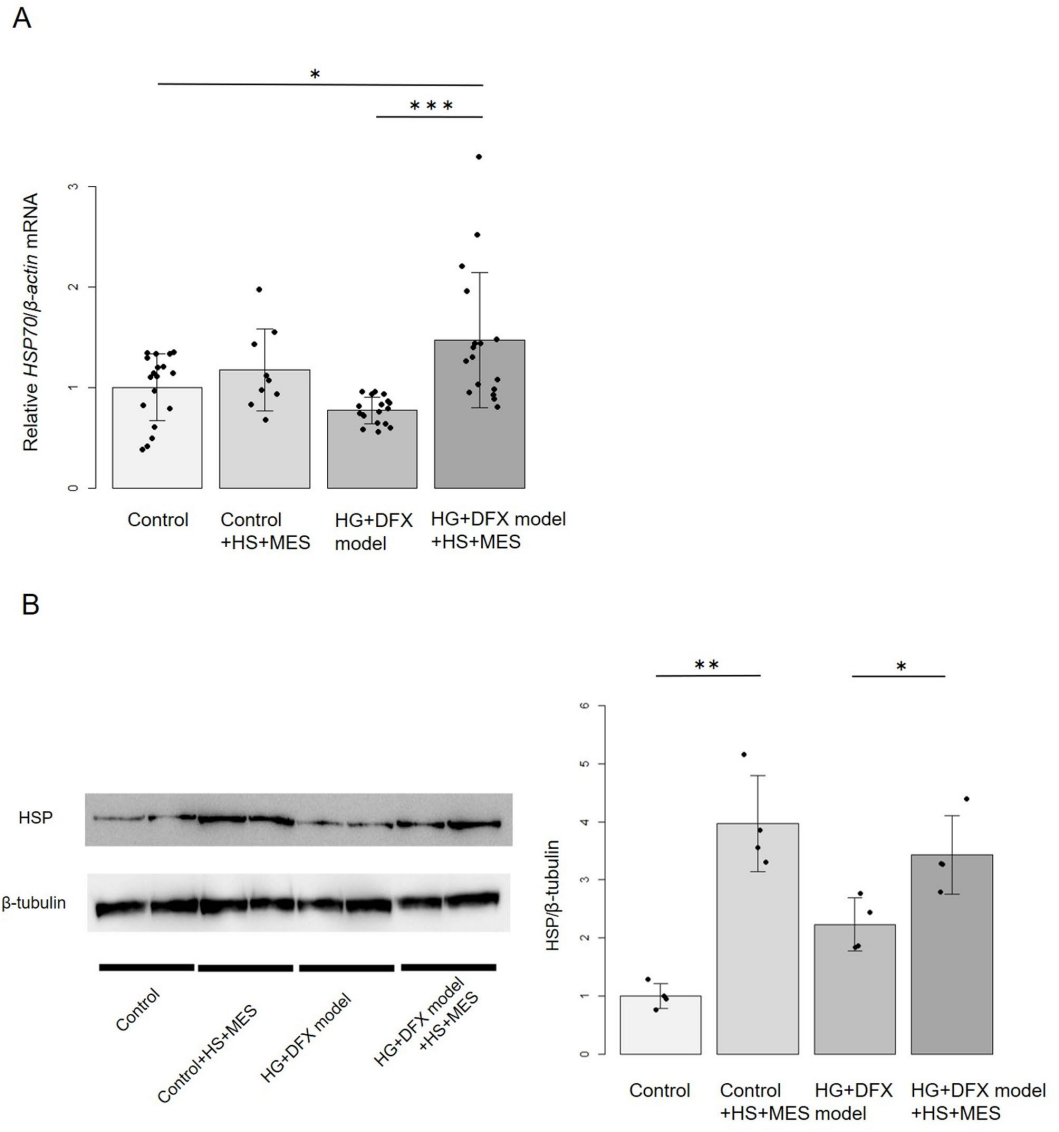
Relative changes in HSP gene and protein by HS + MES therapy. (A) Relative fold change determined by quantitative real-time PCR (qRT-PCR) analysis of *HSP70* in control, control + HS + MES, HG + DFX model, HG + DFX model + HS + MES at 24 h after HS + MES therapy. *HSP70* expression was significantly higher in HG + DFX model + HS + MES group compared to HG + DFX model group. All data were normalized with *β-actin* expression and are given as relative to the control. Data are expressed as means ± standard deviation. *p < 0.05, **p < 0.001, Tukey–Kramer test. Control (*n* = 18), Control་HS + MES (*n* = 9), HG + DFX model (*n* = 18), HG + DFX model ་HS + MES (*n* = 17). (B) Analysis of HSP expression by Western blotting at 6 h after HS + MES therapy. Relative density of HSP was normalized by β-tubulin and compared in each group. HSP expression was significantly higher in HG + DFX model + HS + MES group compared to HG + DFX model group. Data are expressed as means ± standard deviation. *n* = 4/group; *p < 0.05, **p < 0.0001, Bonferroni test. HSP, Heat shock protein; HS, Heat shock therapy; MES, mild electrical stimulation; HG, High glucose; DFX, Deferoxamine mesylate salt

HSP70 is a member of the heat shock protein family that is robustly expressed in RPE cells. Given that HSP70 is upregulated in response to laser photocoagulation and has been recognized as a potential therapeutic target for various diseases [8, 14], we focused on evaluating its expression in this study. mRNA expression of *HSP70* was significantly higher in HG + DFX model + HS + MES group compared to HG + DFX model group 24 h after the treatment (Fig. 1A). There were no significant differences in *HSP70* expression between the control group and the control + HS + MES group. Furthermore, no significant difference in *HSP70* expression was observed between the groups 48 h after treatment (Additional Figure S3A). *HSP70* expression was induced 24 h after HS + MES treatment in the HG + DFX model group. Western blotting analysis demonstrated that HS + MES treatment significantly increased HSP expression in both the control and HG + DFX model groups (Fig. 1B). Quantitative analysis of the western blot bands revealed that HSP expression was significantly higher in the control + HS + MES group compared to the control group. HSP expression was also significantly higher in HG + DFX model + HS + MES group compared to HG + DFX model group. Therefore, HS + MES treatment induced HSP protein expression in both the control and HG + DFX model groups. These results indicate that HS + MES can promote the expression of HSP.

### Effects of HS + MES on gene expression of VEGF and inflammatory cytokines and protein expression of VEGF in HG + DFX model

**Fig. 2:**
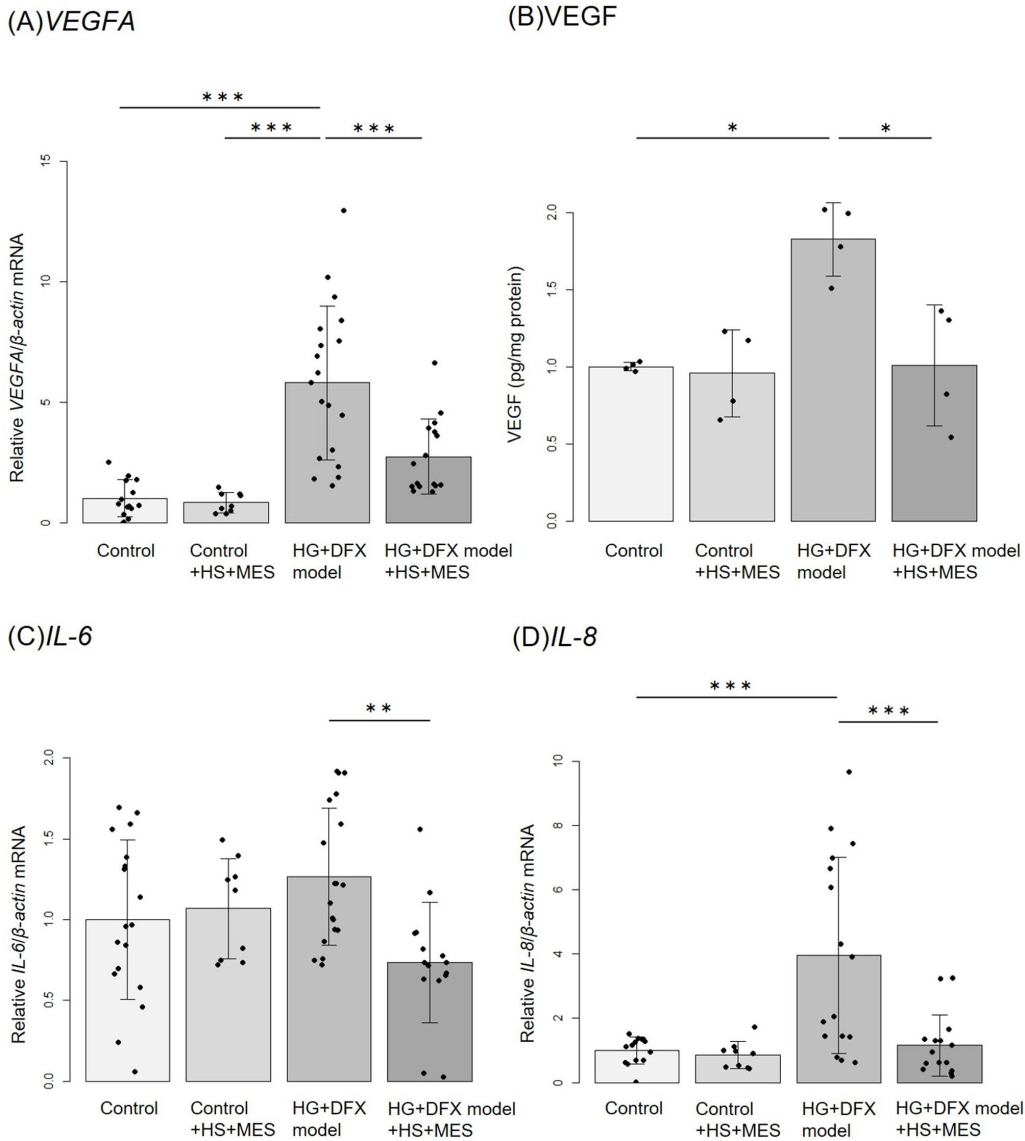
Relative changes in cytokines by HS + MES therapy. (A) Relative fold change determined by quantitative real-time PCR (qRT-PCR) analysis of VEGF gene in control, control + HS + MES, HG + DFX model, HG + DFX model + HS + MES at 24 h after HS + MES therapy. *VEGFA* expression was significantly higher in HG + DFX model group compared to the control group. *VEGFA* was significantly lower in HG + DFX model + HS + MES group compared to HG + DFX model group. All data were normalized with *β-actin* expression and are given as relative to the control. Control (*n* = 16), Control་HS + MES (*n* = 9), HG + DFX model (*n* = 19), HG + DFX model་HS (*n* = 16). (B) Quantitative determination of VEGF protein by ELISA at 24 h after HS + MES therapy. Protein levels of VEGF were significantly higher in HG + DFX model group, while VEGF levels were also significantly lower in HG + DFX model + HS + MES group compared to HG + DFX model group. *n* = 4/group. (C, D) Relative fold change determined by quantitative real-time PCR (qRT-PCR) analysis of HS + MES-related and DME-related inflammatory genes in control, control + HS + MES, HG + DFX model, HG + DFX model + HS + MES at 24 h after HS + MES therapy. *IL-8* expression was significantly higher in HG + DFX model group compared to the control group. *IL-6* and *IL-8* expression was significantly lower in HG + DFX model + HS + MES group compared to HG + DFX model group. All data were normalized with *β-actin* expression and are given as relative to the control. (C) Control (*n* = 18), Control་HS + MES (*n* = 9), HG + DFX model (*n* = 19), HG + DFX model་HS + MES (*n* = 15). (D) Control (*n* = 14), Control་HS + MES (*n* = 9), HG + DFX model (*n* = 16), HG + DFX model་HS + MES (*n* = 15). Data are expressed as means ± standard deviation. *p < 0.05, **p < 0.01, ***p < 0.001, Tukey–Kramer test. HS, Heat shock therapy; MES, mild electrical stimulation; HG, High glucose; DFX, Deferoxamine mesylate salt; ELISA, Enzyme-Linked Immunosorbent Assay

*VEGFA* expression was significantly higher in HG + DFX model group compared to the control group. *VEGFA* expression was significantly lower in HG + DFX model + HS + MES group compared to HG + DFX model group 24 h after the treatment (Fig. 2A). On the other hand, no significant difference in *VEGFA* expression was observed between the groups 48 h after the treatment (Additional Figure S3B). Protein levels of VEGF were also significantly higher in HG + DFX model group, while VEGF levels were also significantly lower in HG + DFX model + HS + MES group compared to HG + DFX model group (Fig. 2B). Among inflammatory cytokines, interleukin (IL)-6 and IL-8 are significantly elevated in the eyes of patients with diabetic retinopathy [28, 29]. mRNA for *IL-6*,* IL-8*,* HIF-1α*, and Intercellular adhesion molecule (*ICAM*)*-1* was measured. *IL-6* and *IL-8* expressions were significantly lower in HG + DFX model + HS + MES group compared to HG + DFX model group 24 h after the treatment (Fig. 2C and D). On the other hand, no significant difference in *IL-6* and *IL-8* expressions were observed between the groups 48 h after the treatment (Additional Figure S3C and D). Furthermore, there were no significant group differences for *HIF-1α* and *ICAM-1* expression 24 h after the treatment (data not shown; *p* = 0.059 and *p* = 0.40, respectively, ANOVA). Therefore, HS + MES treatment decreases a pro-angiogenic factor and inflammatory cytokines in HG + DFX models that simulate DME.

### HS + MES protected the blood-retinal barrier (BRB) integrity and reduces hyperpermeability in HG + DFX model

**Fig. 3:**
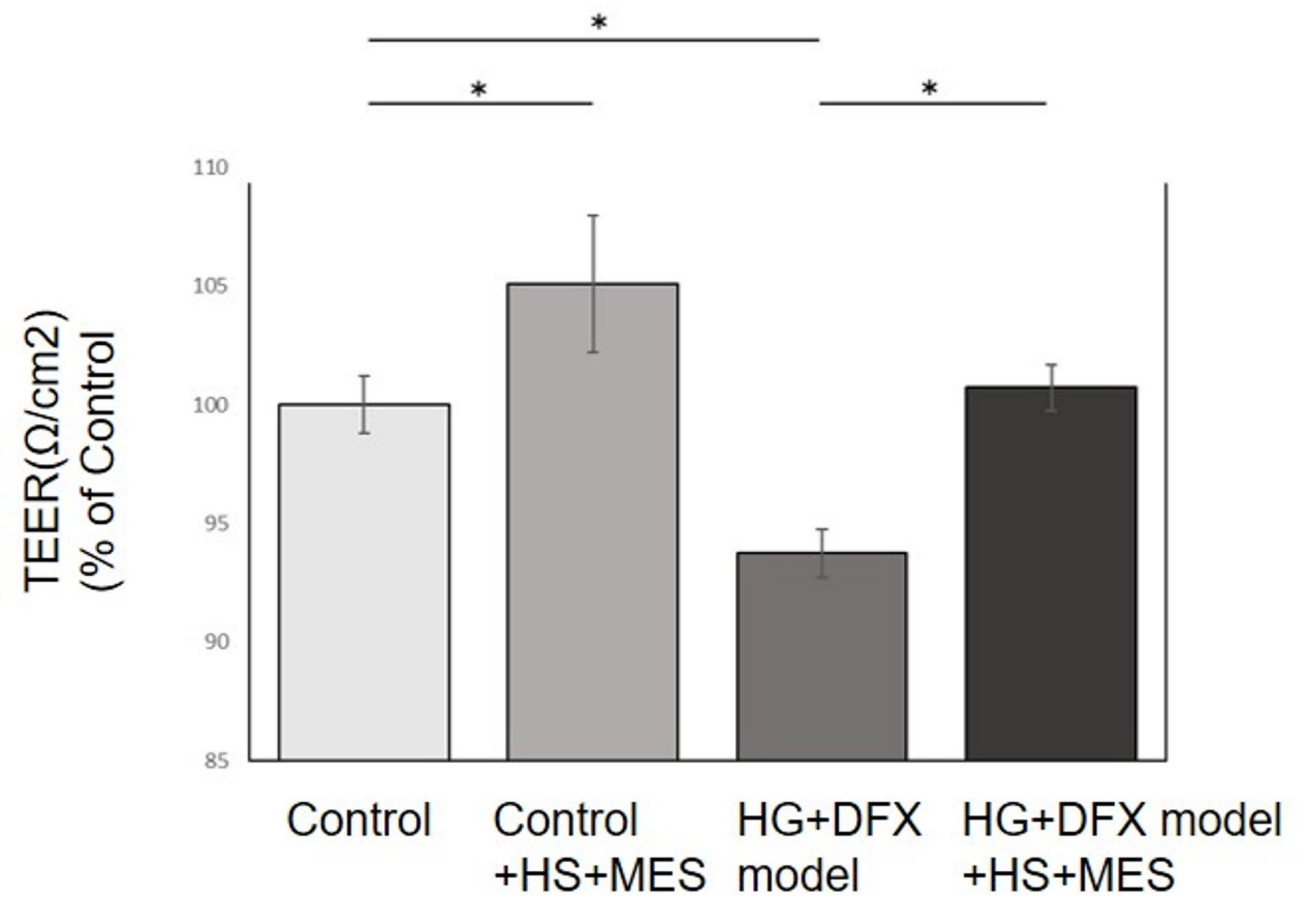
Effect of HS + MES therapy on TEER. TEER values at 24 h after HS + MES therapy. Since the decrease in VEGF expression in HG + DFX model caused by HS + MES treatment was confirmed at 24 h post-treatment, TEER values were also measured at 24 h post-treatment. TEER values were also measured at 48 h after treatment to evaluate changes over time. In both control and HG + DFX model groups, TEER significantly increased 24 h after HS + MES treatment. Data are expressed as means ± standard deviation. *n* = 4/group; *p < 0.05, Tukey–Kramer test. TEER, Transepithelial electrical resistance; HS, Heat shock therapy; MES, Mild electrical stimulation; HG, High glucose; DFX, Deferoxamine mesylate salt

TEER value representing barrier integrity was significantly lower in HG + DFX model group than the control group. In both control and HG + DFX model groups, TEER significantly increased 24 h after HS + MES treatment (Fig. 3). On the other hand, there were no significant differences in either group at 48 h after HS + MES treatment (Additional Figure S4).

## Discussion

The current study investigated the effect of HS and MES on ARPE-19 cells that simulate DME. As a result, the current study found that combined HS + MES treatment increased HSP expression while suppressing VEGF, IL-6 and IL-8 levels in HG + DFX model. In addition, the combined treatment prevented a decrease in TEER in HG + DFX model, although the effect was transient. This therapeutic effect was achieved without inducing significant cytotoxicity, a finding consistent with previous reports on thermal stimulation therapies like micropulse laser [30, 31].

The induction of HSP appears to be a critical upstream event. Chronic inflammation, a hallmark of diabetes, is known to suppress HSP expression, while HSPs themselves can inhibit inflammatory signaling [21, 32, 33]. Although we could not establish a causal relationship between HS + MES-induced HSP70 upregulation and the reduction of VEGF and inflammatory cytokines, our findings suggest that HS + MES restores a protective cellular mechanism that may contribute to these downstream effects. Given that pro-inflammatory cytokines including VEGF-A are drivers of vascular leakage and inflammation in DR and DME [34–37], HS + MES treatment has a potential for clinical application and may represent a promising treatment option for DME that is poorly responsive to conventional therapies such as anti-VEGF and conventional laser therapy.

The current study investigated the impact of HS + MES treatment on the integrity of the BRB in HG + DFX model that simulate DME, measured by TEER. Hyperglycemia and other factors associated with DR can impair BRB [38]. In addition, VEGF plays an important role in the destruction of BRB and the onset of macular edema, while IL-6 may contribute to outer BRB dysfunction [39, 40]. Indeed, inner BRB dysfunction is the primary cause of DME pathogenesis. The breakdown of retinal vascular endothelial cells drives vascular leakage and accumulation of exudates [38, 41]. We focused on RPE cell line incubated by high glucose media as a disease model because micropulse laser specifically targets pigmented structures— that is RPE in retina. In the current study, HS + MES treatment significantly increased TEER values at 24 h post-treatment, suggesting a protective effect on barrier function. However, this improvement was transient and disappeared by the 48 h timepoint. The transient improvement in TEER indicates an increase in HSP, as well as the suppression of VEGF and inflammatory cytokines. This transient effect underscores the need for further optimization. Fine-tuning treatment parameters, such as frequency and duration of application, is necessary to achieve sustained therapeutic benefits.

From a clinical perspective, these findings are highly relevant. The current standard of treatment for DME, intravitreal anti-VEGF injections, is invasive, costly, and associated with treatment burdens and risks [4, 7]. Furthermore, a significant portion of patients show a suboptimal response to anti-VEGF therapy alone [6]. A non-invasive treatment like HS + MES could serve as a valuable adjunctive therapy. By targeting both VEGF and inflammatory pathways, it could potentially reduce the required frequency of anti-VEGF injections, thereby lowering treatment costs, minimizing injection-related complications, and improving disease management for patients who respond poorly to conventional treatments.

In conclusion, our current study suggests that HS and MES treatment has great potential as a new therapeutic approach for managing DR and DME. Future research should focus on elucidating the precise mechanisms by which HS and MES treatment influences these conditions, optimizing treatment parameters, and evaluating its clinical efficacy in animal and human trials.

## Limitations

There are several limitations in this study. The study used an in vitro model of ARPE-19 cells. This model lacks the complexity of the human retina, such as the interplay of different cell types, extracellular matrix, neurovascular networks, blood flow and immune system responses. Furthermore, as an immortalized cell line, ARPE-19 cells may not fully recapitulate the physiology of primary human RPE cells. These factors limit the direct translatability of our findings and underscore the necessity of validating this therapy in appropriate animal models of DME.

Another limitation of this study is that the causal relationship between HSP upregulation and its downstream effects has not yet been demonstrated. Future studies using HSP70 inhibitors will be necessary to verify this mechanistic link.

Since this study did not include a MES-only group, it is difficult to clearly distinguish the contribution of MES alone. While the combination of HS and MES was effective, the specific contribution of MES requires further investigation with inclusion of a MES-only group.

The scope of our molecular investigation was focused. We primarily assessed HSP70, VEGFA, IL-6, and IL-8. The treatment’s effect on other HSP family members, a broader array of cytokines, and other critical cellular pathways (e.g., apoptosis, cell migration, specific signaling cascades) remains to be explored. A more comprehensive mechanistic analysis is needed to fully understand how HS + MES exerts its protective effects.

Pending positive results from preclinical studies, it will be essential to conduct small clinical trials in human patients. The goal will be to test the safety, tolerability and initial efficacy of HS + MES in the treatment of DR and DME. These studies should monitor visual acuity, central retinal thickness and any adverse effects.

## Supplementary Information

Below is the link to the electronic supplementary material.

Supplementary Material 1

## Data Availability

The data that support the findings of this study are available from the corresponding author (R.T.), upon reasonable request.

## Abbreviations

DR: Diabetic retinopathy
DME: Diabetic macular edema
VEGF: Vascular endothelial growth factor
HSP: Heat shock protein
RPE: Retinal pigment epithelial
MES: Mild electrical stimulation
FBS: Fetal bovine serum
DFX: Deferoxamine mesylate salt
HS: Heat shock therapy
LDH: Lactate dehydrogenase
qRT-PCR: Quantitative real-time polymerase chain reaction
ELISA: Enzyme-Linked Immunosorbent Assay
TEER: Transepithelial electrical resistance
IL: Interleukin
HIF: Hypoxia inducible factor
ICAM: Intercellular adhesion molecule
BRB: Blood-retinal barrier
