# Supplementary material for: Combined thermal and mild electrical stimulations modulate heat shock protein and VEGF in retinal pigment epithelial cells under high glucose
Source: BMC Res Notes. 2025 Oct 29;18:459. doi: 10.1186/s13104-025-07539-y (PMC12574004; doi:10.1186/s13104-025-07539-y)
Supplement: Supplementary file 1 — Supplementary Material 1 [file 13104_2025_7539_MOESM3_ESM.docx]

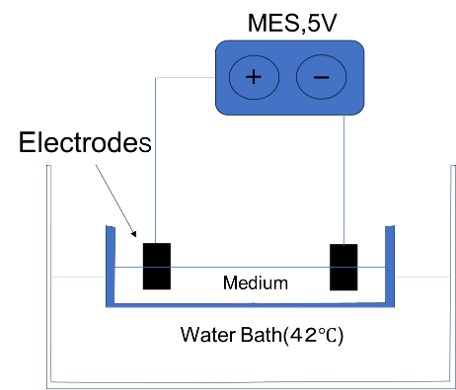


**Additional Figure S1.　Diagram of apparatus for HS+MES treatment**

HS+MES treatment set-up for ARPE-19 cells. HS, Heat shock therapy; MES, Mild electrical stimulation.


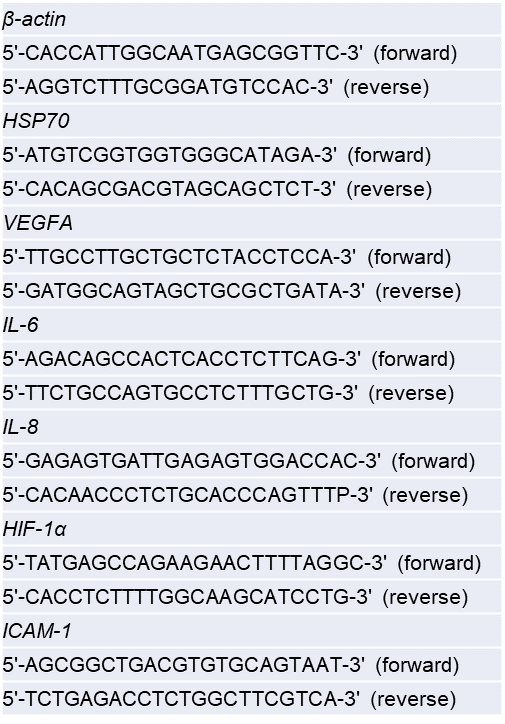


**Additional Table S1.** **Primers used for quantitative real-time PCR**

Control+

HS+MES

HG+DFX model

HG+DFX model

+HS+MES

Cytotoxicity (%)


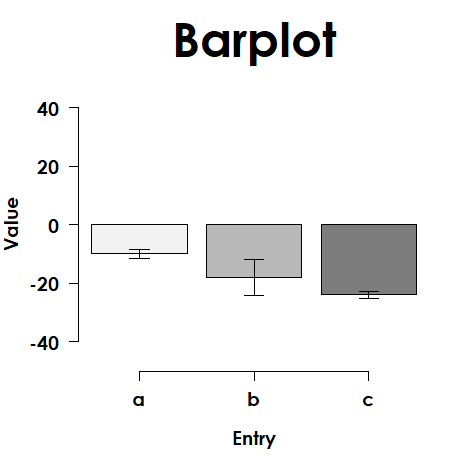


**Additional Figure S2. Assessment of cytotoxicity in HG+DFX models and HS+MES therapy.**

Cytotoxicity rate at 24 h after HS+MES therapy. There was no significant difference in cytotoxicity across groups. Data are each presented as the means ± standard deviation. n= 6/group; *p*= 0.77, ANOVA. HG, High glucose; DFX, Deferoxamine mesylate salt; HS, Heat shock therapy; MES, Mild electrical stimulation

**
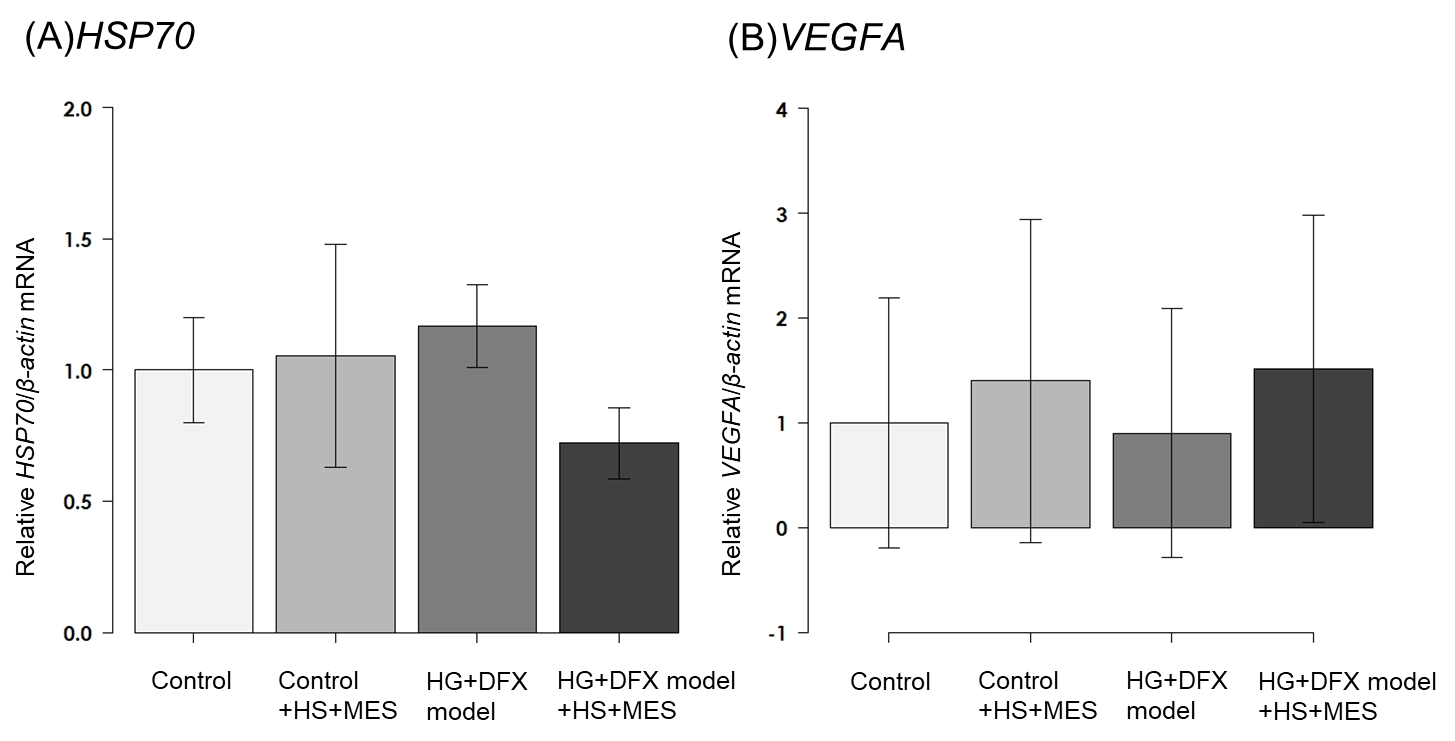
**

**
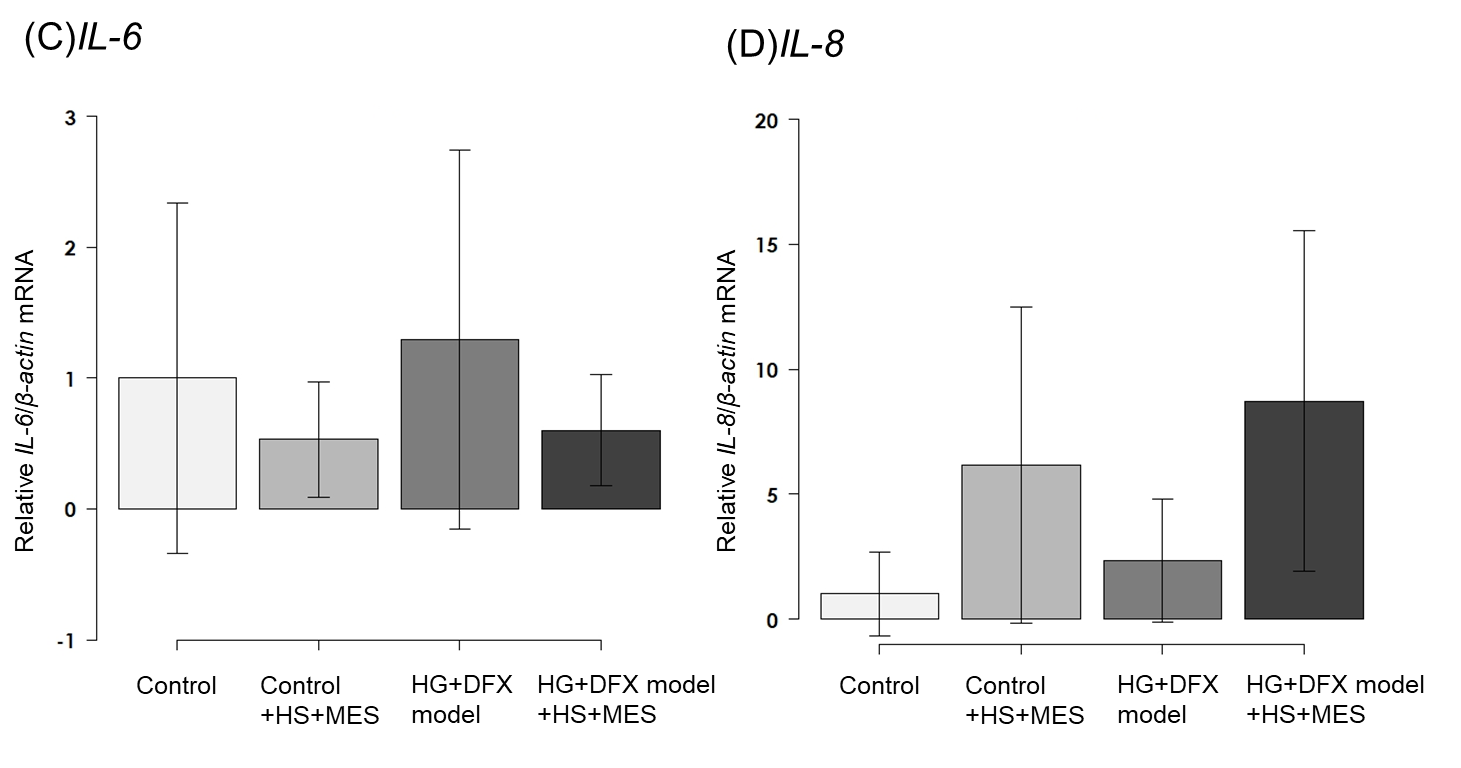
**

**Additional Figure S3. Relative changes in HSP and cytokines by HS+MES therapy.**

(A) Relative fold change determined by quantitative real-time PCR (qRT-PCR) analysis of HSP70 gene in control, control+HS+MES, HG+DFX model, HG+DFX model+HS+MES at 48h after HS+MES therapy. No significant differences in *HSP70* were observed between groups. p= 0.071, ANOVA. All data were normalized with *β-actin* expression and are given as relative to the control. Control (n=6), Control＋HS+MES (n=8), HG+DFX model (n=7), HG+DFX model＋HS (n=6).

(B) Relative fold change determined by quantitative real-time PCR (qRT-PCR) analysis of VEGFA gene in control, control+HS+MES, HG+DFX model, HG+DFX model+HS+MES at 48h after HS+MES therapy. No significant differences in *VEGFA* were observed between groups. p= 0.85, ANOVA. All data were normalized with *β-actin* expression and are given as relative to the control. Control (n=6), Control＋HS+MES (n=8), HG+DFX model (n=7), HG+DFX model＋HS+MES (n=6).

(C,D) Relative fold change determined by quantitative real-time PCR (qRT-PCR) analysis of HS+MES-related and DME-related inflammatory genes in control, control+HS+MES, HG+DFX model, HG+DFX model+HS+MES at 48h after HS+MES therapy. No significant differences in *IL-6* and *IL-8* were observed between groups. p= 0.53 and p= 0.056, respectively, ANOVA. All data were normalized with *β-actin* expression and are given as relative to the control. Control (n=6), Control＋HS+MES (n=8), HG+DFX model (n=8), HG+DFX model＋HS+MES (n=6)

Data are expressed as means ± standard deviation.

HG, High glucose; DFX, Deferoxamine mesylate salt; HSP, Heat shock protein; HS, Heat shock therapy; MES, mild electrical stimulation

**
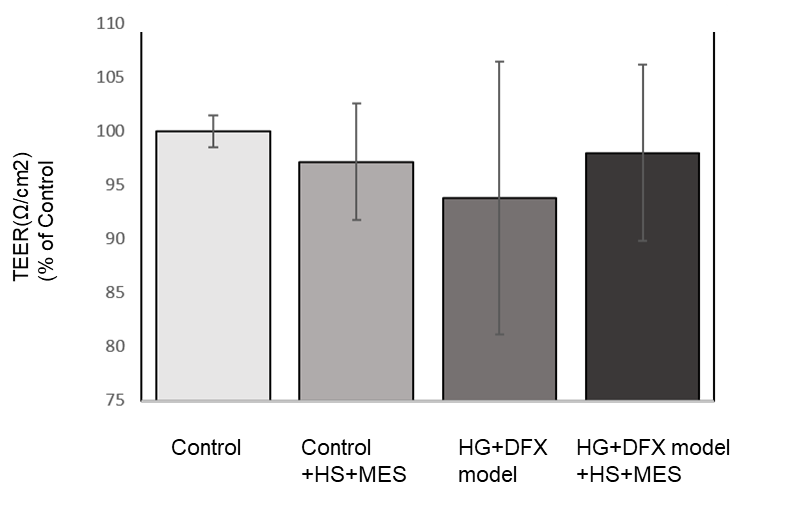
**

**Additional Figure S4. Effect of HS+MES therapy on TEER.**

TEER values at 48h after HS+MES therapy. No significant differences in TEER values were observed between groups. n= 4/group; p= 0.82, ANOVA.

HG, High glucose; DFX, Deferoxamine mesylate salt; TEER, Transepithelial electrical resistance; HS, Heat shock therapy; MES, Mild electrical stimulation

**
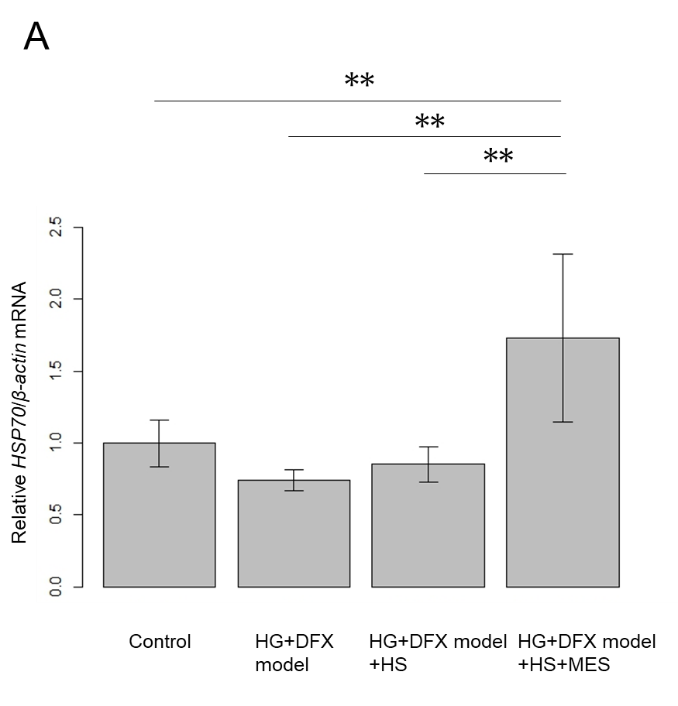
**

**
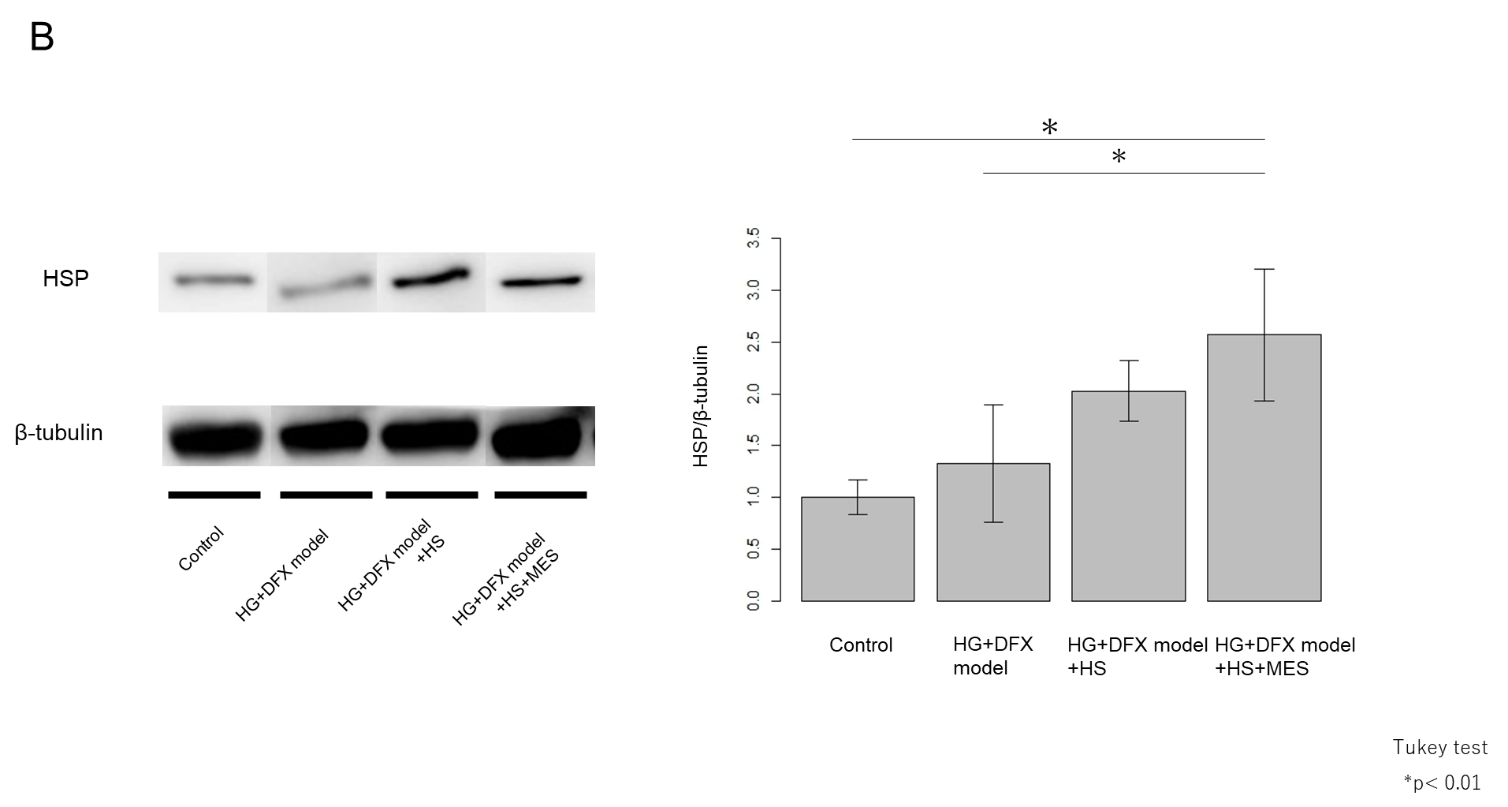
**

**Additional Figure S5. Relative changes in HSP gene and protein by HS or HS+MES therapy.**

(A) Relative fold change determined by quantitative real-time PCR (qRT-PCR) analysis of *HSP70* in control, HG+DFX model, HG+DFX model+HS, HG+DFX model+HS+MES at 24h after HS or HS+MES therapy. *HSP70* expression was significantly higher in HG+DFX model+HS+MES group compared to HG+DFX model group. However, no significant difference in *HSP70* expression was observed between HG+DFX model+HS group and HG+DFX model group. All data were normalized with *β-actin* expression and are given as relative to the control. Control (n=11), HG+DFX model (n=9), HG+DFX model＋HS (n=9), HG+DFX model＋HS+MES (n=8).

(B) Analysis of HSP expression by Western blotting at 6h after HS or HS+MES therapy. Relative density of HSP was normalized by β-tubulin and compared in each group. HSP expression was significantly higher in HG+DFX model+HS+MES group compared to HG+DFX model group. However, no significant difference in HSP expression was observed between HG+DFX model+HS group and HG+DFX model group. Data are expressed as means ± standard deviation. Control (n=3), HG+DFX model (n=6), HG+DFX model+HS (n=6), HG+DFX model+HS+MES (n=6).

Data are expressed as means ± standard deviation. **p* < 0.01, ***p* < 0.001, Tukey–Kramer test. HSP, Heat shock protein; HS, Heat shock therapy; MES, mild electrical stimulation; HG, High glucose; DFX, Deferoxamine mesylate salt.

**
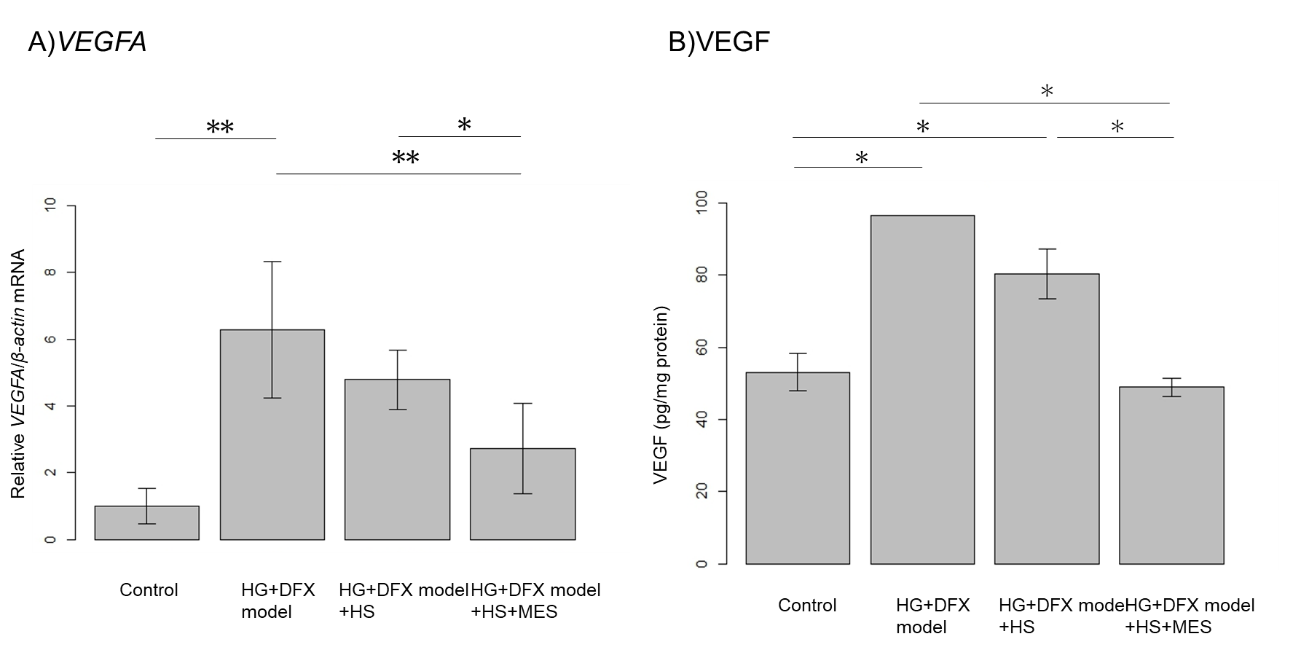
**

**
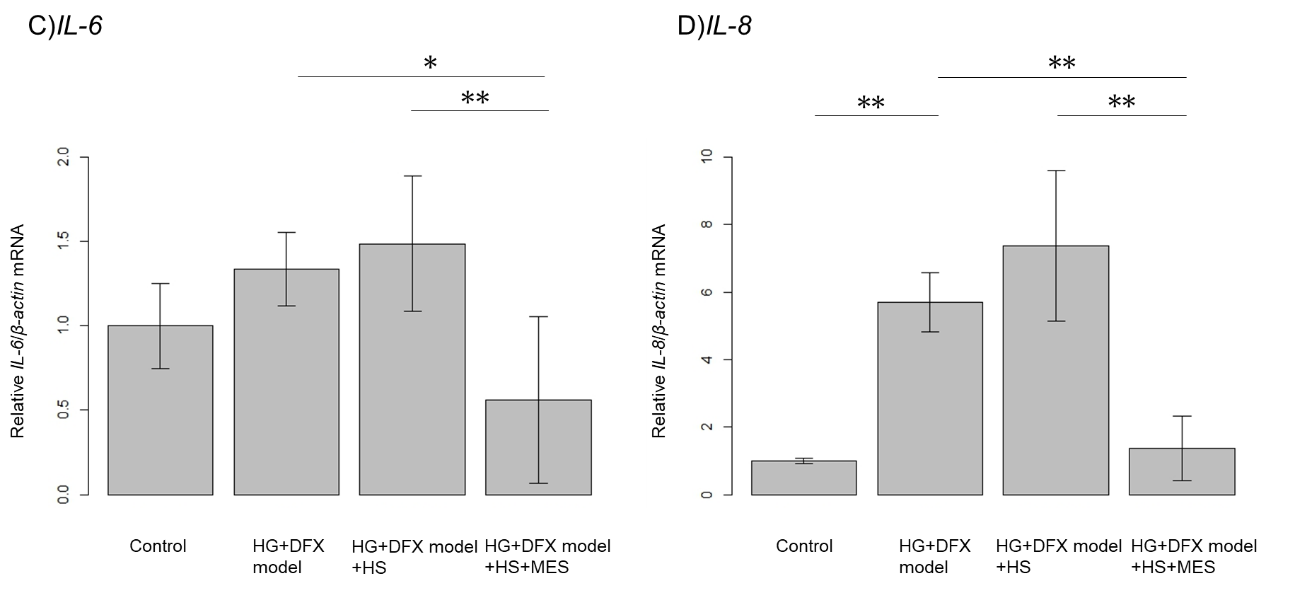
**

**Additional Figure S6. Relative changes in cytokines by HS or HS+MES therapy.**

(A) Relative fold change determined by quantitative real-time PCR (qRT-PCR) analysis of VEGF gene in control, HG+DFX model, HG+DFX model+HS, HG+DFX model+HS+MES at 24h after HS or HS+MES therapy. *VEGFA* was significantly lower in HG+DFX model+HS+MES group compared to HG+DFX model group. However, no significant difference in *VEGFA* expression was observed between HG+DFX model+HS group and HG+DFX model group. All data were normalized with *β-actin* expression and are given as relative to the control. Control (n=10), HG+DFX model (n=9), HG+DFX model+HS (n=9), HG+DFX model+HS (n=7).

(B) Quantitative determination of VEGF protein by ELISA at 24h after HS or HS+MES therapy. Protein levels of VEGF were significantly higher in HG+DFX model group compared to the control group, while VEGF levels were significantly lower in HG+DFX model+HS+MES group compared to HG+DFX model group. However, no significant difference in VEGF levels was observed between HG+DFX model+HS group and HG+DFX model group. n= 4/group.

(C,D) Relative fold change determined by quantitative real-time PCR (qRT-PCR) analysis of HS+MES-related and DME-related inflammatory genes in control, HG+DFX model, HG+DFX model+HS, HG+DFX model+HS+MES at 24h after HS or HS+MES therapy. *IL-8* expression was significantly higher in HG+DFX model group compared to the control group. *IL-6* and *IL-8* expression was significantly lower in HG+DFX model+HS+MES group compared to HG+DFX model group. However, no significant difference in *IL-6* and *IL-8* expression were observed between HG+DFX model+HS group and HG+DFX model group. All data were normalized with *β-actin* expression and are given as relative to the control. (C) Control (n=11), HG+DFX model (n=9), HG+DFX model+HS (n=9), HG+DFX model＋HS+MES (n=5). (D) Control (n=8), HG+DFX model (n=6), HG+DFX model+HS (n=6), HG+DFX model＋HS+MES (n=5)

Data are expressed as means ± standard deviation. **p* < 0.05, ***p* < 0.001, Tukey–Kramer test.

HS, Heat shock therapy; MES, mild electrical stimulation; HG, High glucose; DFX, Deferoxamine mesylate salt; ELISA, Enzyme-Linked Immunosorbent Assay.


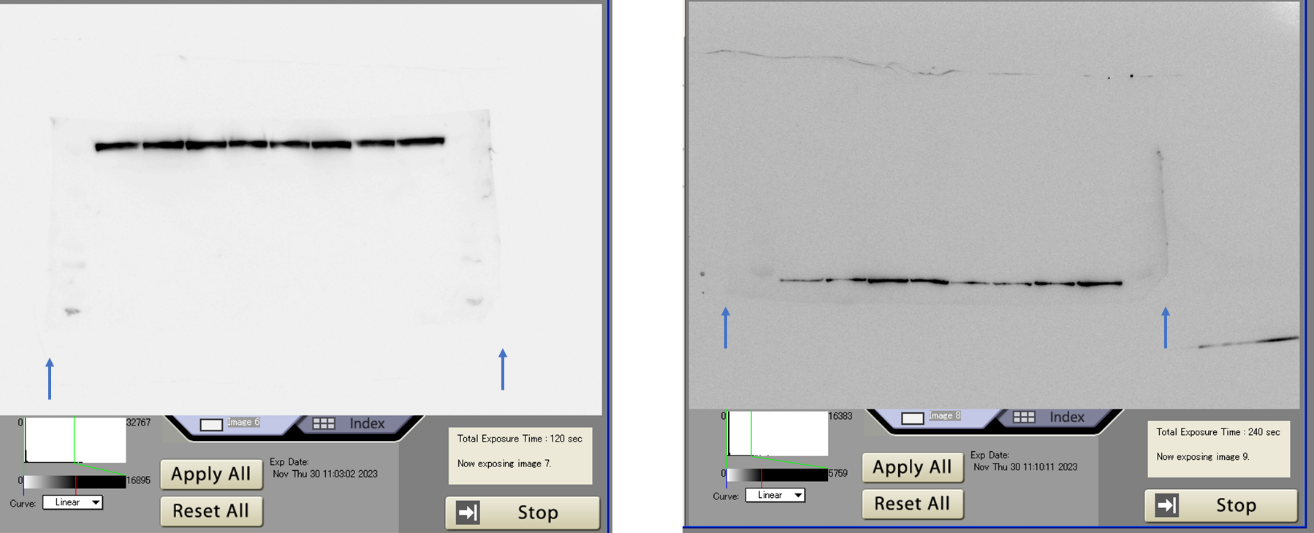


**Additional Figure S7. Unprocessed images of western blotting corresponding to Figure 1B.**

Left: β-tubulin, Right: HSP70. The blue arrow indicates the edge. HSP, Heat shock protein.
